# Principle and design of clinical efficacy observation of extracorporeal cardiac shock wave therapy for patients with myocardial ischemia-reperfusion injury: A prospective randomized controlled trial protocol

**DOI:** 10.1371/journal.pone.0294060

**Published:** 2023-12-08

**Authors:** Xianbin Li, Chaoyue Zhang, Changzhi Liu, Yiming Ma, Yunke Shi, Yujia Ye, Xuejuan Ma, Yixi Liu, Xiang Luo, Fanru Lin, Jincheng Wang, Jifa Tao, Jinping Lun, Hongyan Cai, Zhao Hu

**Affiliations:** 1 Department of Cardiology, The First Affiliated Hospital of Kunming Medical University, Kunming, Yunnan, China; 2 Department of Vascular Surgery, The First Affiliated Hospital of Kunming Medical University, Kunming, Yunnan, China; UN Mehta Institute of Cardiology and Research Center, INDIA

## Abstract

**Background:**

Acute ST-segment elevation myocardial infarction (STEMI) remains a serious life threatening event with a poor prognosis due to myocardial ischemia/reperfusion injury despite coronary revascularization. Extracorporeal cardiac shock wave (ECSW) is a safe, effective and non-invasive new method for the treatment of cardiovascular diseases. The current results show that extracorporeal cardiac shock wave provides a new treatment option for patients with severe and advanced coronary heart disease. However, there are relatively few clinical studies on the application of in vitro cardiac shock waves in patients with myocardial ischemia-reperfusion injury. We hypothesized that extracorporeal cardiac shock therapy would also be effective in reducing clinical endpoints in patients with STEMI reperfusion.

**Objective:**

This study is order to provide a new therapeutic method for patients with myocardial ischemia-reperfusion injury and reveal the possible mechanism of ECSW for ischemia-reperfusion injury.

**Methods and materials:**

CEECSWIIRI is a single-center, prospective randomized controlled trial that plans to enroll 102 eligible patients with acute ST-segment elevation myocardial infarction reperfusion. Eligible patients with STEMI reperfusion will be randomly divided into external cardiac shock therapy (ECSW) trial group and blank control group. The blank control group will receive optimal drug therapy, and the experimental group will receive optimal drug therapy combined with ECSW. The shock wave treatment plan will be 3-month therapy, specifically 1 week of treatment per month, 3 weeks of rest, 3 times of ECSW in each treatment week, respectively on the first day, the third day and the fifth day of the treatment week, lasting for 3 months and follow-up for 2 years. The primary endpoint will be to assess the 2-year improvement in all-cause death, re-hospitalization due to cardiovascular disease, major unintentional cerebrovascular events, including cardiogenic death, myocardial infarction, heart failure, arrhythmia, emergency coronary revascularization, and stroke in patients with STEMI reperfusion. Secondary endpoints will include improvements in angina pectoris, quality of life, cardiac structure and function, coronary microcirculation, and endothelial progenitor cell-derived miR-140-3p in relation to survival outcomes.

**Trial registration number:**

ClinicalTrial.gov.org PRS:NCT05624203; Date of registration: November 12, 2022.

## Introduction

Acute myocardial infarction (AMI) is a serious condition that poses a significant threat to human life and health, resulting in high mortality and morbidity worldwide. At present, the treatment of acute myocardial infarction mainly includes drug therapy, percutaneous coronary intervention (PCI) and coronary artery bypass grafting (CABG), etc. Although the reperfusion and the best drug therapy technologies are mature, the reperfusion process may cause further injury, which is called myocardial ischemia-reperfusion injury (IRI) [1, 2], can cause a series of pathophysiological changes such as myocardial stunning, energy metabolism disorder and microvascular dysfunction [3], and aggravate irreversible damage such as glycogen depletion, nuclear chromatin margination, mitochondrial swelling, sarcolemma rupture [4], however, there is no specific drug in the world to solve this problem.

Extracorporeal cardiac shock wave therapy (ECSW) is a cutting-edge technology that has been developed in the world for more than 20 years. In 2004, ECSW was approved by European CE certification. In the early stage, the efficacy, safety and mechanism of action were discussed in Germany, Japan, Switzerland, Italy and other countries. It is mainly used to treat refractory angina pectoris of coronary heart disease. The mechanism of ECSW is mainly due to the small attenuation, small shear stress, and strong penetration of shock wave in human tissues, resulting in shear stress and cavitation effect in the focal region of the shock wave, triggering microbubbles to form/burst repeatedly within the tissue/cell microenvironment, producing a variety of physical and biological effects [5] and these physical mechanisms trigger a range of biological effects, for example, it can promote the expression of many kinds of cytokines and angiogenic factors, activate related signal transduction pathways, inhibit apoptosis and oxidative stress, and finally increase the number of new blood vessels in the therapeutic area, improve the ischemic state.

The present results suggest that ECSW can effectively treat refractory angina pectoris, significantly improve the clinical symptoms and quality of life, and enhance exercise tolerance in patients with heart failure, providing a new treatment option for patients with severe advanced coronary artery disease [5]. However, clinical studies of ECSW during this period have been relatively few, given that the early phase of acute myocardial infarction is characterized by a high incidence of cardiovascular events. In 2018, Japanese scholars reported that 17 patients with acute myocardial infarction underwent three ECSWS within 48–72h after direct PCI, and echocardiography, cardiac magnetic resonance and left ventricular angiography were used to evaluate left ventricular size and function, and 25 patients were matched as the control group. The 6-month follow-up indicated that LVEF in the ECSW group was significantly improved and the left ventricular diameter showed a decreasing trend, but the difference was not statistically significant [6]. However, due to the small sample size and short follow-up period, whether ECSW can help improve ventricular remodeling in patients with acute myocardial infarction remains to be further studied and explored. We hypothesized that in vitro cardiac shock therapy was effective in reducing the clinical end point in patients with acute ST-segment elevation myocardial infarction reperfusion.

Therefore, we designed this prospective randomized controlled clinical trial to evaluate the clinical efficacy and safety of in vitro cardiac shock therapy for patients with myocardial ischemia-reperfusion injury, with a view to provide a novel therapy for these patients.

## Materials and methods

### Study design

This is a single-center, prospective randomized controlled trial to investigate the clinical efficacy of in vitro cardiac shock wave therapy in patients with myocardial ischemia-reperfusion injury, the difference in the level of endothelial progenitor cell derived miR-140–3p between the shock wave treatment group and the control group, and the relationship between the expression level of miR-140–3p and clinical efficacy and prognosis. In order to provide a new therapy for patients with myocardial ischemia-reperfusion injury.

**Fig 1:**
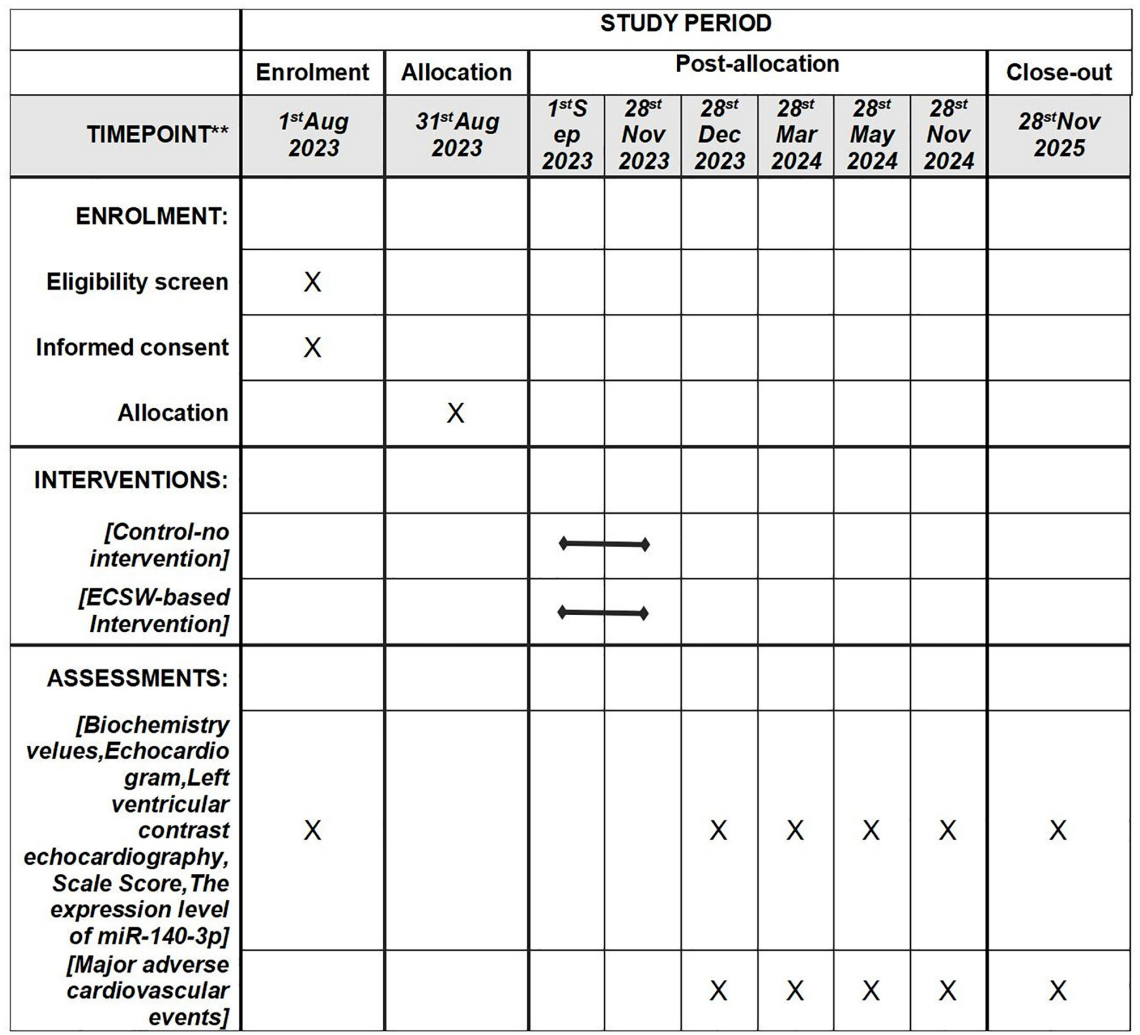
SPIRIT schedule of enrolment, intervention, and assessment of the outcomes. **List specific timepoints in this row.

**Fig 2:**
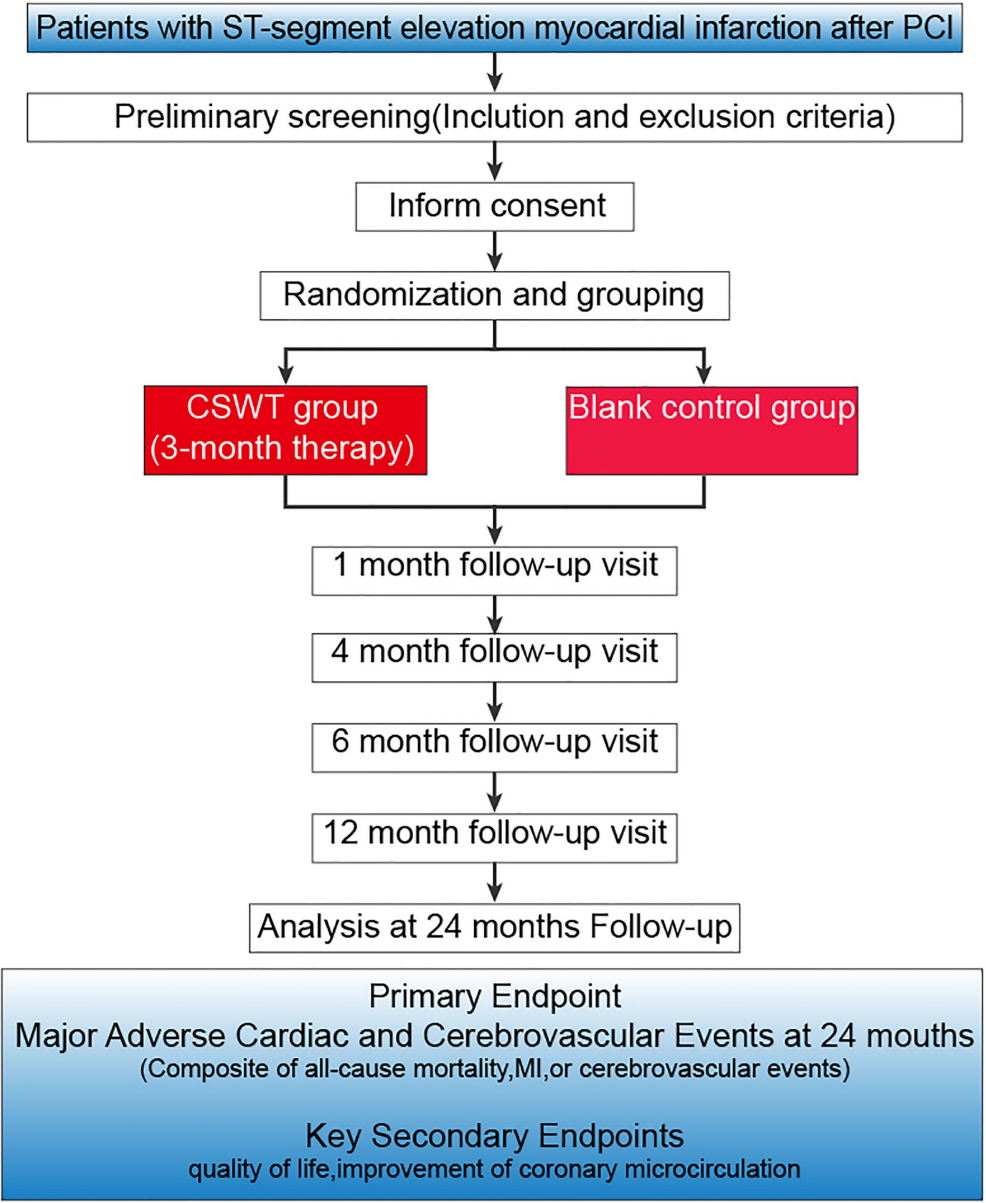
Study flowchart.

Starting on August 1, 2023, all eligible participants will be invited to participate in the study, and a thorough explanation of the study by using an information sheet will be provided by the researcher(Fig 1). The study design has been represented in Fig 2. Patients with acute ST-segment elevation myocardial infarction reperfusion who meet the inclusion criteria and sign informed consent will be randomly divided into extracorporeal cardiac shock therapy (ECSW) trial group and blank control group, and will be followed for 2 years. The primary endpoint will be to assess the 2-year improvement in all-cause death, re-hospitalization due to cardiovascular disease, major unintentional cerebrovascular events, including cardiogenic death, myocardial infarction, heart failure, arrhythmia, emergency coronary revascularization, and stroke in patients with acute ST-segment elevation myocardial infarction undergoing reperfusion. Secondary endpoints will include improvements in angina pectoris, quality of life, cardiac structure and function, coronary microcirculation, and endothelial progenitor cell-derived miR-140–3p in relation to survival outcomes. The protocol requires each patient to provide informed consent before initiating any research procedure. This study is registered with Clinicaltrials.gov (identifier: NCT05624203).

### Participants and informed consent

This study will enroll 102 patients with first-ever acute ST-segment elevation myocardial infarction who were treated with Percutaneous Coronary Intervention (PCI) within 12 hours of onset according to current guidelines for revascularization, and no major adverse clinical events occurred in the previous 6 months, with specific study inclusion and exclusion criteria are shown in Table 1. Eligible candidates will be randomized to receive treatment at the First Affiliated Hospital of Kunming Medical University. Patients who meet the inclusion criteria will be contacted by one of the investigators to confirm their willingness to participate in the trial and arrange a baseline assessment, during which written informed consent will also be obtained. All patients will be informed that they can withdraw from the trial at any time.

**Table 1:** List of inclution and exclution criteria.

Inclution criteria	Exclution criteria
⇒ Age ≥ 18 years. ⇒ First diagnosis of acute ST-segment elevation myocardial infarction, coronary angiography showed moderate to severe coronary artery stenosis, PCI was performed within 12 hours of onset according to current guidelines, and postoperative hemo-dynamics were stable. ⇒ CCS angina pectoris class II or above, NYHA heart function class I-III. ⇒ Imaging examination [stress echocardiography and(or) stress myocardial perfusion imaging] showed objective evidence of reversible myocardial ischemia. ⇒ Voluntary participation, cooperation with treatment and follow-up, signing informed consent form.	⇒ Unprotected severe left main disease. ⇒ Left ventricular systolic function is impaired with hemodynamic instability. ⇒ Poor acoustic window of ultrasound examination caused by chronic obstructive pulmonary disease, pulmonary bullae, false breast implantation or other reasons. ⇒ Combined with malignant tumor of chest. ⇒ Pregnancy. ⇒ Skin ulceration or infection in the treatment area. ⇒ NYHA heart function class IV. ⇒ Acute myocarditis, pericarditis, moderate or large pericardial effusion, infections endocarditis, deep vein thrombosis, intracardiac thrombosis, Severe aortic valve stenosis, aortic aneurysm, thoracic aortic dissection, thoracic aortic aneurysm, post-heart transplantation, post-heart metal valve replacement, pulmonary embolism. ⇒ Patients undergoing thrombolysis and surgical bypass. ⇒ Patients with a history of mental illness, poor compliance and inability to cooperate.

### Randomisation and blinding

Patients with acute ST-elevation myocardial infarction reperfusion who meet the inclusion criteria and sign an informed consent will be randomized by coin toss. With the coin heads up, patients with acute ST-elevation myocardial infarction reperfusion will be assigned to the extracorporeal shock therapy group. With the coin heads down, patients with acute ST-elevation myocardial infarction reperfusion will be assigned to a blank control group. The test group and the control group will be divided into 1:1 proportional grouping. In order to ensure blinding, the details of the random assignment will not be given to the investigator. In addition, Shock wave treatment practitioners and evaluators will be completely separated.

### Study intervention

**Fig 3:**
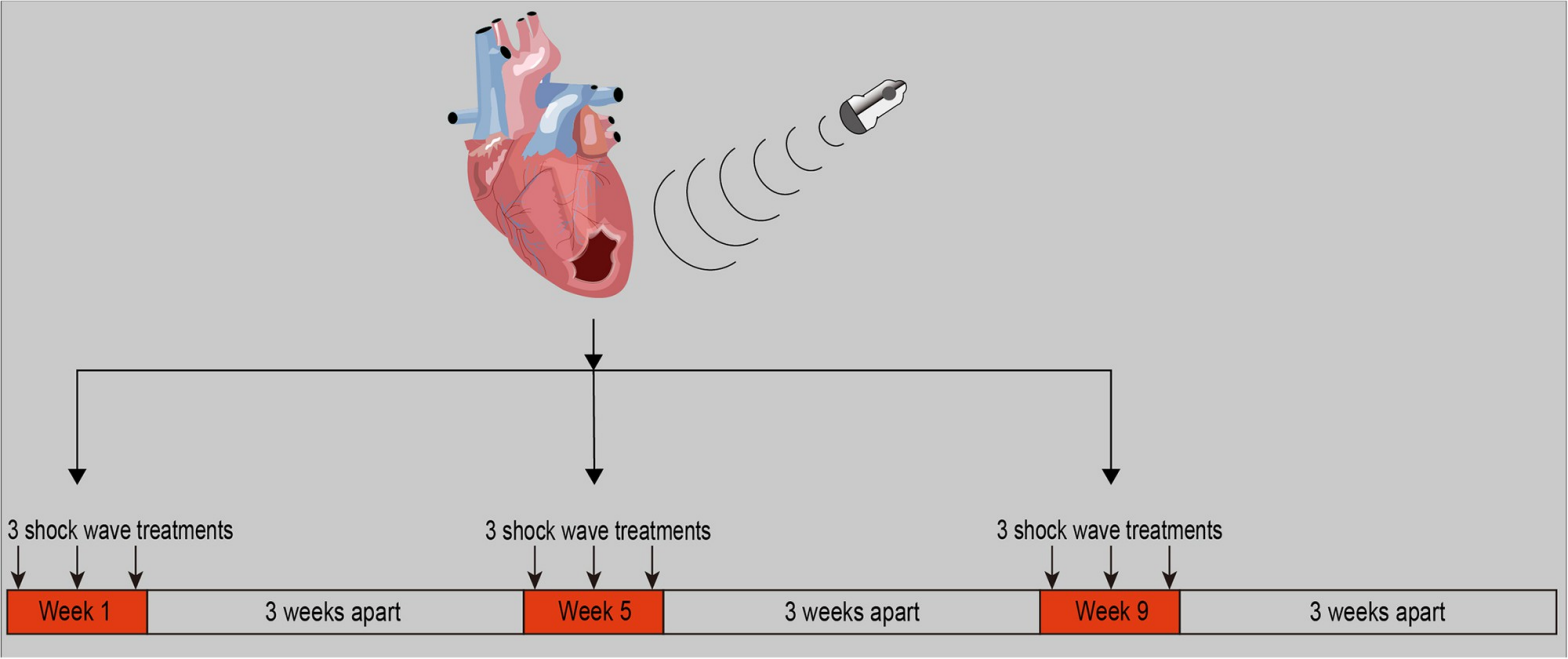
Flow chart of extracorporeal cardiac shock wave therapy. Republished from PLOS ONE under a CC BY license, with permission from Hongyan Cai, original copyright 2023.

This study strictly followed the Helsinki Protocol and Good Clinical Practice (GCP) guidelines, and both the control group and the study group received standard treatment as background treatment. The control group received standard treatment for acute myocardial infarction as background therapy, while the experimental group will be given extracorporeal cardiac shock wave therapy 2–3 days after PCI for acute myocardial infarction in addition to standard therapy as background treatment. The treatment course is 3 months, as shown in Fig 3. Nine treatments were completed within a span of three months as one treatment course. Each month, one week was dedicated to treatment, followed by three weeks of rest. During each treatment week, three sessions of extracorporeal cardiac shock wave will be performed on the 1st, 3rd, and 5th days. This treatment schedule was followed for a total duration of three months.

**Fig 4:**
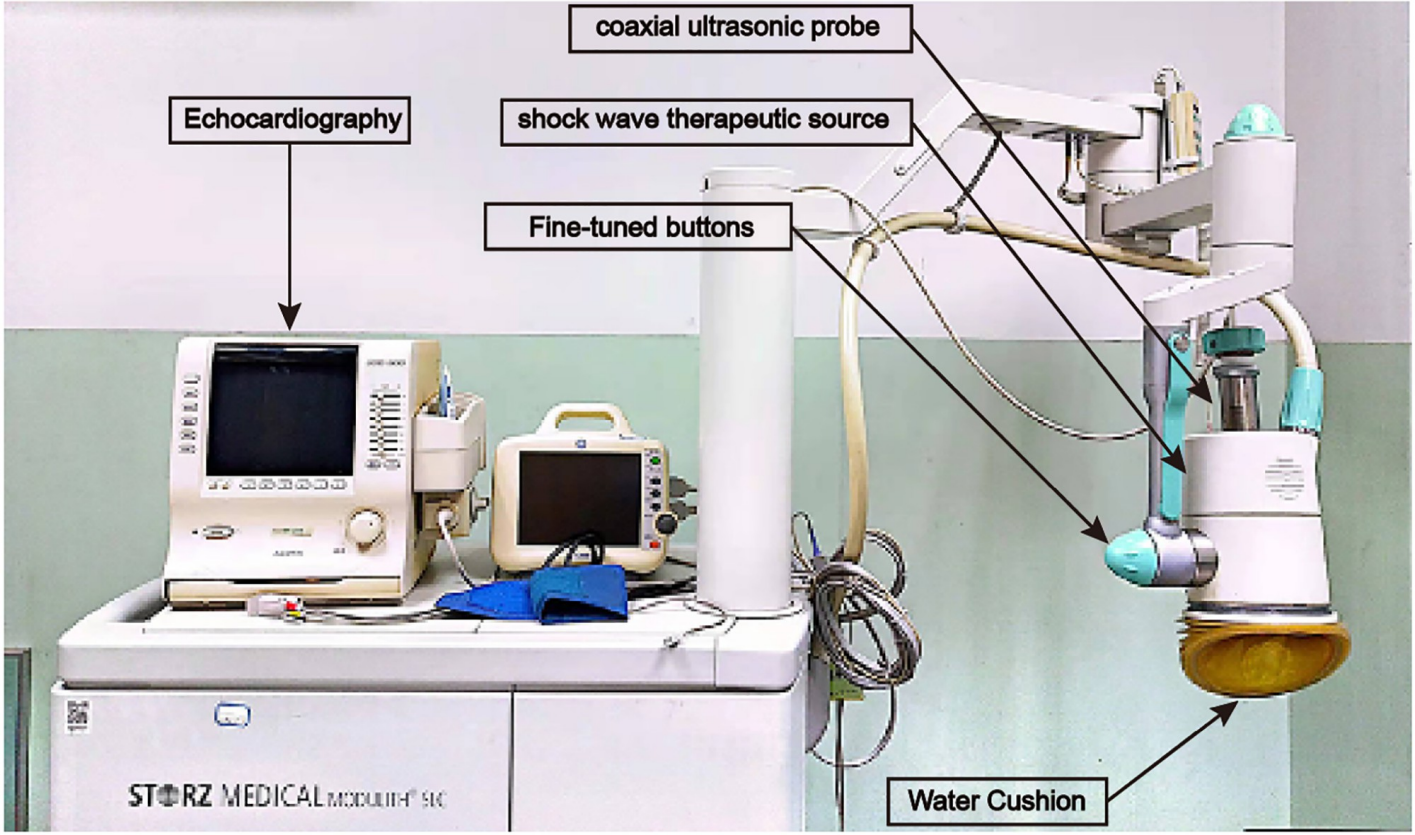
The machine is equipped with a shoch wave therapeutic source and coaxial ultrasonic probe. The SW generator is attached to the chest wall of the patient when used. The SW pulse is easily focused on the ischemic myocardium under the guidance of echocardiography. There is no need of sedatives or anesthesia. Republished from PLOS ONE under a CC BY license, with permission from Hongyan Cai, original copyright 2023.

The experimental group meeting the inclusion and exclusion criteria will be treated by the external cardiac shock therapy instrument shown in Fig 4 in accordance with standard operating procedures, specifically to determine the treatment target myocardium: according to the conventional left ventricular myocardium will be divided into 17 segments, through load ultrasound or load nuclide myocardial perfusion imaging, combined with ECG, coronary angiography and other results, to determine the therapeutic bullsey-eye muscle segments. Vital signs monitoring: During treatment, patients will be required to lie in a quiet position, and 12-lead combined electrocardiogram (ECG) will be recorded, connected to ECG, blood pressure and oxygen saturation monitoring. Setting the height of the water sac: Adjust the height of the water sac (positioning) according to the location of the target myocardium before positioning, and then move the ultrasonic probe down and slightly protruding from the surface of the water sac to achieve clear positioning. After positioning, it is appropriate to increase the height of the water sac until the patient’s chest wall is lightly touched but not compressed, in order to reduce the energy attenuation in the process of seismic wave conduction. Airborne ultrasonic detection of bull’s eye muscle segments: When the probe moves down and touches the chest wall, the left ventricular long axis image will be displayed first, and then the ultrasonic adjustment button will be rotated clockwise to display the left ventricular short axis, four chambers and two chambers of the left heart section in turn. When more than two sections lock the same segment together, the positioning will be regarded as successful. Energy control: Raise the probe after positioning so as not to interfere with shock wave release. Then press the shock wave releaser, starting from a small energy level of 0.8 (equivalent to 0.024 mJ/mm2). The successful release is marked by the occurrence of “Da, da” sound consistent with the patient’s heart rate. Every 200 points is one frequency cycle. If there is no special, the energy level can be gradually adjusted to level 3 (0.09 mJ/mm2); Fine-tuning of treatment points in the target area: under normal circumstances, we will line a combination of 9 points between 1 ∼ 0 ∼ -1 or 2 ∼ -2 for each area, and issue 200 pulses at each point, giving a total of 1800 pulses (9 × 200). Monitoring during treatment: ask patients about their symptoms and observe their vital signs and electrocardiogram. In case of discomfort or changes in electrocardiogram, lower the energy level first and continue to observe, treat as appropriate, record and analyze the causes in detail.

### Laboratory assessments

Assessment the expression level of miR-140–3p in exosomes derived from endothelial progenitor cells (EPCs).

First, density gradient centrifugation will be used to separate endothelial progenitor cells from peripheral blood of the shock wave intervention group and the blank control group. Under aseptic conditions, 6 mL of heparin anticoagulant peripheral blood will be collected from the peripheral veins of the selected subjects, and the specimens will be processed within 4 hours after collection. The 6ml of blood taken will be added to a centrifuge tube containing 6mL of the lymphocyte separation solution, giving it a 1:1 volume ratio. Centrifuge at 2500rmp for 30min. After centrifugation, the liquid will be divided into four layers: plasma layer, mononuclear cell layer, separation liquid layer and erythrocyte and granulocyte layer. The mononuclear cell layer of the narrow band of the white cloud layer is taken. The extracted mononuclear cell layers will be co-incubated with corresponding primary antibodies (CD31, CD34, CD133, and VEGFR2) and FitC-labeled secondary antibodies, and then flow cytometry will be used for sorting and identification of endothelial progenitor cells.

Secondly, exosomes in EPCs will be extracted and identified. EPCs cell fluid sorted by flow cytometry will be collected. Exosomes will be purified from cell fluid using an ExoQuick-TC Kit(System Biosciences Inc, USA) according to the manufacturer’s instructions. In short, the cell solution will be fully mixed with 1/5 volume exosome separators, will be incubated overnight at 4°C, then the mixture will be centrifuged at 1,500×g at 4°C for 30min, the exosomes will be collected, and the exosome particles at the bottom of the tube will be re-suspended in 100ul PBS solution and stored at −80°C for further study. Exosomes will be identified by nanoparticle tracking analysis (NanoFCM, N30E), exosome labeling expression, and transmission electron microscopy (Hitachi, HT-7700, Japan). Particle size distribution and concentration information will be detected by nanoparticle tracking analysis. Exosome markers CD9, CD63, CD81 and Calnexin will be identified by Western blotting. The ultrastructure of exosomes will be observed with transmission electron microscopy. The protein concentration of exosomes will be tested with a BCA kit from Thermo Fisher Science.

Finally, the expression level of miR-140–3p in the extracted exosomes will be measured, and total RNA will be extracted from the exosomes using Trizol^™^ reagent (Invitgen USA). RNA reverse transcription will be performed with Mir-X miRNA first Chain synthesis kit (Japan Takara), and miRNAs will be quantified with Mir-X miRNA qRT-PCR TB Green kit (Japan Takara). After purification, the extracted RNA will be reverse-transcribed into cDNA using the Takara PrimeScrip^™^RT kit (Takara Japan) with gDNA eraser as a template. Using TB Green PreMix Ex Taq^™^II(Tli RNAseH Plus)(Japan Takara) for a gene-specific primer pair polymerase chain reaction (miR-140–3p Forward:5 ‘-TACCACAGGGTAGAACCACGG-3’), SqRT-PCR will be performed on QuantStudio6Flex Real-time quantitative Polymerase Chain Reaction System (QuantStudio6Flex Real-Time PCRsystem, USA). The reaction system is as follows: At 95°C for 30s, 95°C for 40 cycles for 5s, and 60°C for 34s, the relative expression level of miR-140–3p will be detected by 2−ΔΔCt. U6 (Forward:5 ‘-CTCGCTTCGGCAGCACA-3’ Reverse:5 ‘-AACGCTTCACGAATTTGCGT-3’) will be used as an endogenous control against miR-140–3p.

### Data collection

All necessary data will be collected and recorded in electronic case report forms for this study, which will be mainly obtained from medical records(such as electronic or paper medical records), local laboratory testing records and investigator’s evaluation on patients (Table 2). Relevant baseline data will be collected at the time of enrollment in patients with acute ST-segment elevation myocardial infarction reperfusion, mainly including:demography(age, gender, race), medical history, signs, treatment, biochemical values(mainly including serum electrolyte value, serum AST, serum ALT, serum creatinine, serum BUN, serum CK-mb, cTNI, BNP and HsCRP), imaging examination indicators(Echocardiography will be used to measure left ventricular ejection fraction (LVEF), left ventricular end diastolic diameter(LVEDD), global longitudinal strain, myocardial stress changes in each segment and synchrony of myocardial motion, left ventricular contrast echocardiography will assess coronary microcirculation), relevant scale scores(mainly will include 6-minute walk test, Canada Cardiovascular Society (CCS) angina classification, Seattle angina scale (SAQ) score, quality of life scale (SF-8)) and expression level of miR-140–3p in endothelial progenitor cell (EPCs)-derived exosomes in peripheral blood;Biochemical values, imaging parameters, scores of relevant scales, expression level of miR-140–3p in endothelial progenitor cell (EPCs)-derived exosomes and major adverse cardiovascular events (which will include all-cause death, readmission due to cardiovascular disease, cardiac death, myocardial infarction, heart failure, arrhythmia, emergency coronary revascularization and stroke) will be collected in the test group and control group at 1, 4, 6, 12 and 24 months.

**Table 2:** Study plan and time points of key assessments.

Item	Enrolment	time(month)				
		1	4	6	12	24
Screening patients based on inclusion and exclusion criteria	√					
Informed consent‡	√					
Demographics	√					
Medical history	√					
Physical examination	√					
Vital signs	√					
Biochemistry values**	√	√	√	√	√	√
Echocardiogram¶	√	√	√	√	√	√
Left ventricular contrast echocardiography††	√	√	√	√	√	√
Scale Score‡‡	√	√	√	√	√	√
The expression level of miR-140–3p§	√	√	√	√	√	√
MACE+		√	√	√	√	√

^‡^Informed consent form should be signed by the patients at enrollment.

**Biochemistry values including serum electrolyte values, serum AST and serum ALT, serum creatinine, serum BUN, serum CK-MB, cTNI, BNP and HsCRP will be collected if available as per clinical practice.

^¶^Echocardiography will be used to measure left ventricular ejection fraction (LVEF), left ventricular end-diastolic diameter (LVEDD), global longitudinal strain, changes in myocardial stress at various segments, and synchrony of myocardial motion.

^††^Left ventricular contrast acoustics will be used to assess the coronary microcirculation.

^‡‡^ Relevant scales will include the 6-minute walking test, the Canadian Cardiovascular Society (CCS) Angina Scale, the Seattle Angina Scale (SAQ) score, and the Quality of Life Scale (SF-8).

^§^Determination of EPC-derived miR-140–3p will be used to reveal its association with survival outcomes in patients with acute ST-segment elevation myocardial infarction reperfusion.

^+^ MACE: Major adverse cardiovascular events.

AST, Aspartate aminotransferase; ALT, alanine aminotransferase; BUN, blood urea nitrogen; CK-MB, Creatine kinase isoenzyme; cTNI, cardiac troponin I; BNP, Brain natriuretic peptide; HsCRP, Hypersensitive C-reactive protein;

### Primary outcomes

The primary endpoint of this study is 2-year all-cause death, rehospitalization due to cardiovascular disease, major adverse cardiovascular and cerebrovascular events, including cardiac death, myocardial infarction, heart failure, arrhythmia, emergency coronary revascularization, and stroke;

### Secondary outcomes

Time of first occurrence of cardiovascular death, such as repeat myocardial infarction, arrhythmia, or heart failure; improvements in angina pectoris, quality of life, cardiac structure, cardiac function, and coronary microcirculation in patients, as well as expression levels of miR-140–3p derived from endothelial progenitor cells (EPCs).

### Potential risk management

Participants will be informed that there will be no cost for participating in this study, and it will not replace the health services provided by the healthcare professionals of the study hospital. The participants who refuse to continue the study for any given reason will be allowed to pull back from the study without any cost, and withdrawing from the study would not affect the amount of care they receive from the hospital. In addition, during the process of in vitro cardiac shock therapy, we will ask patients about their symptoms and observe their vital signs and electrocardiogram. In case of discomfort or changes in electrocardiogram, we can first reduce the energy level and continue to observe, as appropriate, record and analyze the reasons. A significant advantage of CSWT is its non-invasive nature. A study that monitored CSWT patients for up to 6 years found no adverse events, such as severe arrhythmias, heart failure, respiratory failure, bleeding, embolism, or cardiogenic shock, during treatment. Furthermore, no shockwave-related adverse events were detected during the 6-year follow-up period. It has also been reported that patients are at risk of potential lung injury during treatment. This may be accompanied by mild pain and skin redness or ecchymosis in the treated area. However, these skin changes are mild and typically disappear after 1 week. Finally, if participants’ health or personal privacy is compromised by participating in this study, please inform the researchers and we will take necessary medical measures. According to the relevant regulations of our country, when the study-based injury occurred, the bid party for this research will bear the corresponding medical expense and provide the corresponding economic compensation for the internal medicine of cardiology.

### Sample size estimation

This study is a randomized controlled trial. The experimental group will be received extracorporeal cardiac shock wave therapy intervention, while the control group will be received conventional treatment for acute myocardial infarction. The left ventricular ejection fraction (LVEF) of the study object is one of the observed outcome indicators. According to the literature review results [6], the average LVEF in the control group is 58.0 ± 11.7 points. It is estimated that LVEF in the shock wave intervention group can be increased by 8.1%, *α* = 0.05, and the assurance is 90%. Calculate the sample size (1) according to the following sample size calculation formula [7]

$$\begin{array}{c} n=\frac{2(Z_{\alpha }+Z_{\beta }{)}^{2}*\sigma^{2}}{\delta^{2}} \end{array}$$ (1)

44 cases can be calculated, considering 1:1 randomization, that is, 44 cases in the intervention group and 44 cases in the control group, and considering 15% will lost to follow-up and refuse to visit, finally, at least 51 subjects in the intervention group and 51 subjects in the control group will be needed, and a total of at least 102 subjects will be included.

### Data analysis

Statistical analysis will be performed using SPSS 26.0, Graphpad Prism Software, and for quantitative data, the results will be presented as mean ± standard deviation (x ± s) or median (m) and interquartile range (IQR), between-group comparisons will use Welch’s t-test or Wilcoxon’s rank-sum test; categorical data will be presented as frequencies or percentages, and between-group comparisons will use the chi-square test or Fisher’s exact probability method. Univariate and multivariate associations of ECSW, clinical, echocardiographic, location and degree of coronary artery disease and expression level of endothelial progenitor cell-derived miR-140–3p variables with the cardiovascular events were assessed with Cox’s proportional hazard modelling using the time from inclusion in the study to the date of event. Kaplan–Meyer analysis was performed to estimate probabilities of cardiovascular events. Receiver operating characteristic curves (ROC analyses) were performed to compare the predictive value of the expression level of endothelial progenitor cell-derived miR-140–3p, ECSW, global longitudinal strain (GLS), LVEF and Location and degree of coronary artery disease. with P values less than 0.05 considered statistically significant. The study results will be released to the participating physicians, referring physicians, patients and the general medical community.

## Discussion

The prevalence of ST-segment elevation myocardial infarction (STEMI) is increasing year by year with the aging population in China, China PEACE analyzed 13815 inpatient medical records of 162 secondary and tertiary hospitals randomly selected from 31 provinces, autonomous regions, and municipalities directly under the central government in mainland China, and found that the number of patients hospitalized for ST-segment elevation myocardial infarction (STEMI) per 100,000 people in China increased year by year from 2001 to 2011 [8], The rate of STEMI hospitalizations increased from 3.7 per 100,000 in 2001 to 8.1 per 100,000 in 2006 and 15.8 per 100,000 in 2011, based on natural population estimates. The China-PEACE [9] study showed that the early recurrence rate of myocardial infarction after discharge in China AMI patients was high, with a recurrence rate of myocardial infarction within 1 year of 2.5%, of which 35.7% occurred within 30 days after discharge. The 1-year mortality rate of patients with recurrent myocardial infarction increased by 25.42 times, and the 1-year mortality rate of patients with early recurrent myocardial infarction was the highest (53.5%). Although the best drugs and reperfusion and other treatment technologies are constantly mature, the reperfusion process may cause further injury, Tomas Jernberg [10] et al. found that in the clinical trial of coronary artery reperfusion strategy, the 1-year cardiovascular mortality rate was about 13%, and there was no specific drug to solve this problem in the world at present.

In order to reduce myocardial ischemia-reperfusion injury, some promising multi-target cardioprotective therapies have been proposed [11]. Drug therapy, ischemic conditioning therapy, physical hypothermia therapy, and cell therapy have been applied in clinical trials, but the therapeutic effect in some cases is not ideal [12]. In recent years, stem cell therapy has been considered one of the effective methods to treat various diseases, especially cardiovascular diseases [13]. Many studies have shown that mesenchymal stem cells (MSCs) [14], embryonic stem cells (ESCs) [15], endothelial progenitor cells (EPCS) [16], and induced pluripotent stem cells (IPSCs) [17] have repair and regeneration effects on cardiovascular diseases through myocardial or intravenous injection. However, stem cell therapy has been clinically limited due to low graft cell survival, potential immunogenicity and tumorigenesis, and concerns about cell storage and transport difficulties [18].

In recent years, stem cell-derived exosomes have been observed to have more advantages than cell therapy, which can replicate many of the same cardioprotective effects of stem cells and allow local and distant metastasis of biological contents [19, 20]. In addition, exosomes can be modified by pretreatment or artificial fabrication to make them more effective for targeted therapy [21, 22]. Exosome secretion may be influenced by the internal environment and external stimuli. Extracorporeal cardiac shock wave therapy has been shown to induce post-ischemic cardiac exosome release, thereby improving myocardial fibrosis and left ventricular function [23]. Vicencio et al. found that exosomes extracted from remote ischemic preconditioning (RIPC) plasma can reduce the infarct size of myocardial ischemia-reperfusion rats [24]. In addition, exosomes secreted by cardiomyocytes under ischemic conditions have also been shown to promote cardiac angiogenesis [25]. Therefore, stimulation of exosomal-producing cells may be a viable alternative to exert cardioprotective effects.

Our previous studies confirmed that the cardioprotective effects of Vitro cardiac shock therapy are primarily achieved by promoting the proliferation, mobilization, and activation of endothelial progenitor cells [26]. Subsequent studies further found that ECSW treatment (500 injections at 0.09 mJ/mm2) significantly improved cell viability, anti-apoptosis, migration, and tube formation of EPCs, which may be related to the promotion of downstream p-eNOS and anti-apoptotic protein Bcl-2 expression and inhibition of pro-apoptotic protein Bax and Caspase3 expression through PI3K/AKT and MEK/ERK signaling pathways [27, 28].

Several clinical studies have shown that extracorporeal cardiac shock waves have shown good efficacy in refractory angina [6, 29, 30], stable angina [31–33], and ischemic heart failure [34–37]. Our recent studies confirm that ECSW is an effective stimulus for activating the function of exosomes derived from endothelial progenitor cells, ESWT-activated exosomes derived from endothelial progenitor cells (SW-EXO) have anti-apoptosis, anti-oxidative stress, and anti-inflammation effects, and have therapeutic effects on hypoxia/reoxygenation (H/R) injury of cardiomyocytes, this therapeutic effect is mainly due to miR-140–3p, a key cardioprotective mediator in exosomes, which mainly regulates the PTEN/PI3K/AKT pathway to suppress cell damage by acting in concert with a range of related proteins. These findings provide a novel mechanism for the role of ECSW-induced endothelial progenitor-derived exosomes (EPCS-EXO) in cardioprotection and provide an alternative cell-free approach for myocardial IRI [38].

However, given the high incidence of cardiovascular events in the early stage of acute myocardial infarction, there are relatively few clinical studies on ECSW in this period. In addition, there are few reports on the efficacy and prognosis of miR-140–3p in EPC-derived exosomes and myocardial ischemia-reperfusion injury. The purpose of this study is to observe the clinical efficacy of extracorporeal cardiac shock wave treatment in patients with myocardial ischemia-reperfusion injury, the difference in the level of endothelial progenitor cell-derived miR-140- 3p between the shock wave treatment group and the control group, and the relationship between the level of endothelial progenitor cell-derived miR-140- 3p and clinical efficacy and prognosis, to provide a new evidence-based treatment scheme for patients with myocardial ischemia-reperfusion injury and further improve the prognosis of STEMI patients in the reperfusion era.

During the 24-month study period, the main expected obstacle of this study is the compliance of patients with regular outpatient follow-up, which may lead to missing data and inaccurate results. To avoid this situation, we will provide some items with a higher cost such as extracorporeal cardiac shock wave therapy free of charge during the follow-up process. In addition, the investigators shall thoroughly inform the patients participating in the study of all details of this study to ensure the satisfactory compliance of patients.

## Limitations of the study

Given that the inclusion criteria were limited to patients who underwent percutaneous coronary intervention (PCI) to recanalize the vessel within 12 hours of disease onset and were hemodynamically stable after surgery, our results may not be applicable to all patients who underwent PCI to recanalize the vessel. In addition, the in vitro application of SWT to treat ischemic hearts has several limitations: (a) a small acoustic window, (b) a limited accessible treatment area limited to the anterior myocardium, and (c) the risk of potential lung injury.

## Supporting information

S1 Checklist SPIRIT-checklist. (DOC)

S1 File Study protocol approved by the ethics committee. (DOCX)

S2 File external funder confirming the funding award. (PDF)

S3 File Study protocol approved by the ethics committee in Chinese. (PDF)

S4 File Study protocol approved by the ethics committee in English. (DOC)
