# Supplementary material for: Principle and design of clinical efficacy observation of extracorporeal cardiac shock wave therapy for patients with myocardial ischemia-reperfusion injury: A prospective randomized controlled trial protocol
Source: PLoS One. 2023 Dec 8;18(12):e0294060. doi: 10.1371/journal.pone.0294060 (PMC10707494; doi:10.1371/journal.pone.0294060)
Supplement: S1 File — (DOCX) [file pone.0294060.s002.docx]

**Clinical efficacy of extracorporeal cardiac shock wave therapy for myocardial ischemia-reperfusion injury: a prospective randomized controlled trial**

**Introduction**

Acute myocardial infarction (AMI) is a serious killer threatening human life and health, with high mortality and morbidity worldwide. At present, the treatment of acute myocardial infarction mainly includes drug therapy, percutaneous coronary intervention (PCI) and coronary artery bypass grafting (CABG), etc. Although the reperfusion and the best drug therapy technologies are mature, the reperfusion process may cause further injury, which is called myocardial ischemia-reperfusion injury (IRI)^1,2^, can cause a series of pathophysiological changes such as myocardial stunning, energy metabolism disorder and microvascular dysfunction^3^, and aggravate irreversible damage such as glycogen depletion, nuclear chromatin margination, mitochondrial swelling, sarcolemma rupture^4^, however, there is no specific drug in the world to solve this problem.

Extracorporeal cardiac shock wave therapy (ECSW) is a cutting-edge technology developed in the world for more than 20 years. In 2004, ECSW was approved by European CE certification. In the early stage, the efficacy, safety and mechanism of action were discussed in Germany, Japan, Switzerland, Italy and other countries. It is mainly used to treat refractory angina pectoris of coronary heart disease. The mechanism of ECSW is mainly due to the small attenuation, small shear stress, and strong penetration of shock wave in human tissues, resulting in shear stress and cavitation effect in the focal region of the shock wave, triggering microbubbles to form/burst repeatedly within the tissue/cell microenvironment, producing a variety of physical and biological effects^5^ and these physical mechanisms trigger a range of biological effects, for example, it can promote the expression of many kinds of cytokines and angiogenic factors, activate related signal transduction pathways, inhibit apoptosis and oxidative stress, and finally increase the number of new blood vessels in the therapeutic area, improve the ischemic state.

The present results suggest that ECSW can effectively treat refractory angina pectoris, significantly improve the clinical symptoms and quality of life, and improve exercise tolerance in patients with heart failure, providing a new treatment option for patients with severe advanced coronary artery disease^5^. However, clinical studies of ECSW during this period have been relatively few, given that the early phase of acute myocardial infarction is characterized by a high incidence of cardiovascular events. In 2018, Japanese scholars reported that 17 patients with acute myocardial infarction underwent three ECSWS within 48-72h after direct PCI, and echocardiography, cardiac magnetic resonance and left ventricular angiography were used to evaluate left ventricular size and function, and 25 patients were matched as the control group. The 6-month follow-up indicated that LVEF in the ECSW group was significantly improved and the left ventricular diameter showed a decreasing trend, but the difference was not statistically significant^6^. However, due to the small sample size and short follow-up period, whether ECSW can help improve ventricular remodeling in patients with acute myocardial infarction remains to be further studied and explored. We hypothesized that in vitro cardiac shock therapy was effective in reducing the clinical end point in patients with acute ST-segment elevation myocardial infarction reperfusion.

Therefore, we designed this prospective randomized controlled clinical trial to evaluate the clinical efficacy and safety of in vitro cardiac shock therapy for patients with myocardial ischemia-reperfusion injury, with a view to providing a novel therapy for patients with myocardial ischemia-reperfusion injury.

**Methodology**

**Study aim and objectives**

This study is order to provide a new therapeutic method for patients with myocardial ischemia-reperfusion injury and reveal the possible mechanism of ECSW for ischemia-reperfusion injury.

The objectives of the study as follows:

**I.** The primary endpoint of this study is 2-year all-cause death, rehospitalization due to cardiovascular disease, major adverse cardiovascular and cerebrovascular events, including cardiac death, myocardial infarction, heart failure, arrhythmia, emergency coronary revascularization, and stroke;

**II.**Secondary endpoints are improvement in angina, quality of life, cardiac structure and function, coronary microcirculation, and expression level of endothelial progenitor cell-derived miR-140-3p.

**Research hypothesis**

We hypothesized that extracorporeal cardiac shock therapy would also be effective in reducing clinical endpoints in patients with STEMI reperfusion.

**Study design**

This is a single-center, prospective randomized controlled trial to investigate the clinical efficacy of in vitro cardiac shock wave therapy in patients with myocardial ischemia-reperfusion injury, the difference in the level of endothelial progenitor cell derived miR-140-3p between the shock wave treatment group and the control group, and the relationship between the expression level of miR-140-3p and clinical efficacy and prognosis. In order to provide a new therapy for patients with myocardial ischemia-reperfusion injury. Participants will be patients with first-time acute ST-segment elevation myocardial infarction who have undergone percutaneous coronary intervention (PCI) within 12 hours of onset according to current guidelines for revascularization and have had no major adverse clinical events in the past 6 months. A thorough explanation of the study by using the information sheet (Appendix A) will be provided to all eligible participants and informed consent also will be obtained from the participants before the commencement of the study (Appendix B).

**Study setting and subjects**

The study protocol and implementation will have complied with the principles of Declaration of Helsinki^7^. Firstly, The study will be approved by the Ethics Review Committee of the First Affiliated Hospital of Kunming Medical University. Permission will also be obtained from the Director of the First Affiliated Hospital of Kunming Medical University. Secondly, the purpose of the study, the data collection procedures, the potential risk and benefits, the maintenance of confidentiality, and the voluntary basis of participation will be clearly explained to the participants. Participants will be informed that they have the choice to withdraw from the study at any time if they choose to do without penalty or negative consequence of current health services.

Thirdly, the informed consent will be provided in written and oral form when the research personnel approaches the potential participants. The detailed information and explanation of the nature of the study, the right of participants to withdraw from the study at any time, the anonymity and confidentiality of the participants, contact details of the researcher which participants may contact to obtain further information if they need will be included in the subject information sheet. Fourthly, to maintain the anonymity of the participants, they will be asked not to write their name in the questionnaire, however, the code number will be provided by the researcher. All data will be kept by the principal investigator in a password-protected computer and a locked cabinet to ensure the confidentiality of participants. The participants will be informed that all personal data will only be accessed by the principal investigator and all the collected data will be destroyed five years after the study completion.

This study will be conducted in the First Affiliated Hospital of Kunming Medical University. The study site is a 4,000-bed hospital serving more than 40 million people in Yunnan Province and neighboring regions. Most acute myocardial infarction patients in Yunnan province will receive treatment at the hospital. Study participants will be recruited from study hospital wards. The intervention will be performed at the hospital's cardiology outpatient clinic. The hospital provides multidisciplinary services, including cardiologists, doctors, nurses and other health care workers. The flow chart for the main study is shown in Appendix C.

**Inclusion and exclusion criteria**

The eligibilities of participants are; ≥18 years old; Acute ST-segment elevation myocardial infarction was diagnosed for the first time, and coronary angiography indicated moderate to severe coronary artery stenosis. PCI was performed within 12 hours of the onset of the disease according to the current guidelines, and the postoperative hemodynamics were stable; CCS angina pectoris grade Ⅱ and above, NYHA cardiac function grade I-Ⅲ; Imaging examination [stress echocardiography and/or stress myocardial perfusion imaging] suggested objective evidence of reversible myocardial ischemia; Voluntary participation, able to cooperate with treatment and follow-up, signed informed consent.

Exclusion criteria will include: unprotected severe left main disease; Left ventricular systolic function was impaired and hemodynamic instability was observed. Poor sound window in ultrasound examination due to chronic obstructive pulmonary disease, bullae, pseudobreast implantation or other reasons; Combined with breast malignancy; Pregnancy; Treatment area skin ulceration or infection; NYHA cardiac function grade Ⅳ; Acute myocarditis, pericarditis, moderate or large pericardial effusion, infective endocarditis, deep vein thrombosis, intracardiac thrombus; Severe aortic stenosis, aortic aneurysm, thoracic aortic dissection, thoracic aortic aneurysm, heart transplantation, metal heart valve replacement, pulmonary embolism; Patients undergoing thrombolysis and surgical bypass; Patients with a history of mental illness, poor compliance and inability to cooperate.

**Randomization**

Patients with acute ST-elevation myocardial infarction reperfusion who met the inclusion criteria and signed an informed consent will be randomized by coin toss. With the coin heads up, patients with acute ST-elevation myocardial infarction reperfusion will be assigned to the extracorporeal shock therapy group. With the coin heads down, patients with acute ST-elevation myocardial infarction reperfusion will be assigned to a blank control group.The test group and the control group will be divided into 1:1 proportional grouping. In order to ensure blinding, the details of the random assignment will not be given to the investigator. In addition, Shock wave treatment practitioners and evaluators will be completely separated.

**Sample size estimation**

This study is a randomized controlled trial. The experimental group will be received extracorporeal cardiac shock wave therapy intervention, while the control group will be received conventional treatment for acute myocardial infarction. The left ventricular ejection fraction (LVEF) of the study object is one of the observed outcome indicators. According to the literature review results, the average LVEF in the control group is 58.0 ± 11.7 points. It is estimated that LVEF in the shock wave intervention group can be increased by 8.1%,α= 0.05, and the assurance is 90%. Calculate the sample size according to the following sample size calculation formula


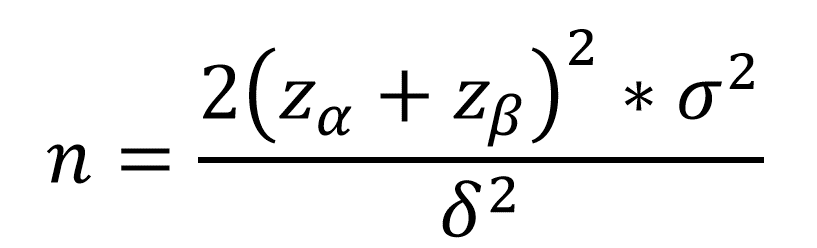


44 cases can be calculated, considering 1:1 randomization, that is, 44 cases in the intervention group and 44 cases in the control group, and considering 15% will lost to follow-up and refuse to visit, finally, at least 51 subjects in the intervention group and 51 subjects in the control group will be needed, and a total of at least 102 subjects will be included.

**Study intervention**

The experimental group will be given extracorporeal cardiac shock wave therapy 2 - 3 days after PCI for acute myocardial infarction, and the treatment course is 3 months. 9 treatments were completed within 3 months as 1 treatment course, 1 week per month, 3 weeks rest, and 3 times of extracorporeal cardiac shock wave will be performed in each treatment week, respectively on the 1st, 3rd, and 5th days of the treatment week, lasting for 3 months in total; The control group received conventional treatment for acute myocardial infarction.

The experimental group meeting the inclusion and exclusion criteria will be treated by the external cardiac shock therapy instrument in accordance with standard operating procedures, specifically to determine the treatment target myocardium: according to the conventional left ventricular myocardium will be divided into 17 segments, through load ultrasound or load nuclide myocardial perfusion imaging, combined with ECG, coronary angiography and other results, to determine the therapeutic bullsey-eye muscle segments. Vital signs monitoring: During treatment, patients will be required to lie in a quiet position, and 12-lead combined electrocardiogram (ECG) will be recorded, connected to ECG, blood pressure and oxygen saturation monitoring. Setting the height of the water sac: Adjust the height of the water sac (positioning) according to the location of the target myocardium before positioning, and then move the ultrasonic probe down and slightly protruding from the surface of the water sac to achieve clear positioning. After positioning, it is appropriate to increase the height of the water sac until the patient's chest wall is lightly touched but not compressed, in order to reduce the energy attenuation in the process of seismic wave conduction. Airborne ultrasonic detection of bull's eye muscle segments: When the probe moves down and touches the chest wall, the left ventricular long axis image will be displayed first, and then the ultrasonic adjustment button will be rotated clockwise to display the left ventricular short axis, four chambers and two chambers of the left heart section in turn. When more than two sections lock the same segment together, the positioning will be regarded as successful. Energy control: Raise the probe after positioning so as not to interfere with shock wave release. Then press the shock wave releaser, starting from a small energy level of 0.8 (equivalent to 0.024 mJ/mm2). The successful release is marked by the occurrence of "Da, da" sound consistent with the patient's heart rate. Every 200 points is one frequency cycle. If there is no special, the energy level can be gradually adjusted to level 3 (0.09 mJ/mm2); Fine-tuning of treatment points in the target area: under normal circumstances, we will line a combination of 9 points between 1 ~ 0 ~ -1 or 2 ~ 0 ~ -2 for each area, and issue 200 pulses at each point, giving a total of 1800 pulses (9×200). Monitoring during treatment: ask patients about their symptoms and observe their vital signs and electrocardiogram. In case of discomfort or changes in electrocardiogram, lower the energy level first and continue to observe, treat as appropriate, record and analyze the causes in detail.

**Data collection and follow-up**

All necessary data will be collected and recorded in electronic case report forms for this study, which will be mainly obtained from medical records(such as electronic or paper medical records), local laboratory testing records and investigator’s evaluation on patients. Relevant baseline data will be collected at the time of enrollment in patients with acute ST-segment elevation myocardial infarction reperfusion, mainly including:demography(age, gender, race), medical history, signs, treatment, biochemical values(mainly including serum electrolyte value, serum AST, serum ALT, serum creatinine, serum BUN, serum CK-mb, cTNI, BNP and HsCRP), imaging examination indicators(Echocardiography will be used to measure left ventricular ejection fraction (LVEF), left ventricular end diastolic diameter(LVEDD), global longitudinal strain, myocardial stress changes in each segment and synchrony of myocardial motion, left ventricular contrast echocardiography will assess coronary microcirculation), relevant scale scores(mainly will include 6-minute walk test, Canada Cardiovascular Society (CCS) angina classification, Seattle angina scale (SAQ) score, quality of life scale (SF-8)) (Appendix D) and expression level of miR-140-3p in endothelial progenitor cell (EPCs)-derived exosomes in peripheral blood;Biochemical values, imaging parameters, scores of relevant scales, expression level of miR-140-3p in endothelial progenitor cell (EPCs)-derived exosomes and major adverse cardiovascular events (which will include all-cause death, readmission due to cardiovascular disease, cardiac death, myocardial infarction, heart failure, arrhythmia, emergency coronary revascularization and stroke) will be collected in the test group and control group at 1, 4, 6, 12 and 24 months.

**Data analysis**

Statistical analysis will be performed using SPSS 26.0, Graphpad Prism Software, and for quantitative data, the results will be presented as mean ± standard deviation (x ± s) or median (m) and interquartile range (IQR), between-group comparisons will use Welch's t-test or Wilcoxon's rank-sum test; categorical data will be presented as frequencies or percentages, and between-group comparisons will use the chi-square test or Fisher's exact probability method, time-to-event variables will be estimated using the Kaplan-Meier method, compared using the log-rank or generalized rank-sum test methods, and Cox regression models will be established, with P values less than 0.05 considered statistically significant.

**Ethical consideration**

This study was approved by the Institutional Ethics Committee of Kunming Medical University's first affiliated hospital. This trial protocol had been registered in ClinicalTrials.gov Protocol Registration and Results System (PRS) (NCT05624203). The study protocol adhered to the principles of the Declaration of Helsinki. The approval was also obtained from the study hospital. The purpose of the study, data collection procedures, potential risks and benefits, maintenance of confidentiality, and voluntary basis of participation will be clearly explained to the participants. Written informed consent of the participants will be obtained from them. To ensure the participants’ confidentiality during the study, their names will be coded, and the codes will be stored separately to ensure anonymity. All electronic data will be stored by the principal investigator in a password-protected computer in a secured place. The paper records will be kept by the principal investigator in a locked cabinet. The personal data will only be accessed by the principal investigator. In addition, entered data will be cross checked by a second person. All the data collected will be destroyed 5 years after the study is completed.

**Significance of the study**

This study is expected to provide a new therapeutic approach for patients with myocardial ischemia-reperfusion injury and reveal the possible mechanism of ECSW treatment for ischemia-reperfusion injury.

**Reference list**

1 Eltzschig, H. K. & Eckle, T. Ischemia and reperfusion--from mechanism to translation. *Nat Med* **17**, 1391-1401, doi:10.1038/nm.2507 (2011).

2 Bulluck, H., Yellon, D. M. & Hausenloy, D. J. Reducing myocardial infarct size: challenges and future opportunities. *Heart* **102**, 341-348, doi:10.1136/heartjnl-2015-307855 (2016).

3 Hausenloy, D. J. & Yellon, D. M. Myocardial ischemia-reperfusion injury: a neglected therapeutic target. *J Clin Invest* **123**, 92-100, doi:10.1172/JCI62874 (2013).

4 Heusch, G. Myocardial ischaemia-reperfusion injury and cardioprotection in perspective. *Nature reviews. Cardiology* **17**, 773-789, doi:10.1038/s41569-020-0403-y (2020).

5 He Qing, H. Y., Xu Yawei, et al. Chinese experts on the treatment of coronary heart disease with Zeta Shock Wave, consensus (2022 edition). *Chinese Journal of Cardiovascular Disease* **5**, 1-12, doi:10.3760/cma.j.cn116031.2022.1000105 (2022).

6 Kagaya, Y. *et al.* Low-energy cardiac shockwave therapy to suppress left ventricular remodeling in patients with acute myocardial infarction: a first-in-human study. *Coron Artery Dis* **29**, 294-300, doi:10.1097/MCA.0000000000000577 (2018).

7 Association, W. M. World Medical Association Declaration of Helsinki: ethical principles for medical research involving human subjects. *JAMA* **310(20)**, 2191-2194, doi:10.1001/jama.2013.281053. PMID: 24141714 (2013).

**Appendix A. Subject Information Sheet**

**Clinical efficacy of extracorporeal cardiac shock wave therapy for myocardial ischemia-reperfusion injury: a prospective randomized controlled trial**

Dear participant,

My name is Li Xianbin. I am currently studying for my master's degree in the First Affiliated Hospital of Kunming Medical University. You are invited to participate in a study entitled **" Clinical efficacy of extracorporeal cardiac shock wave therapy for myocardial ischemia-reperfusion injury: a prospective randomized controlled trial"**.

This study will aim to further improve the clinical symptoms and long-term prognosis of patients with myocardial ischemia-reperfusion injury by using extracorporeal shock therapy, improve coronary microcirculation, reverse ventricular remodeling, relieve angina symptoms, improve quality of life and reduce the occurrence of cardiovascular adverse events by providing free extracorporeal shock therapy based physical therapy intervention. The results of this study may provide a new therapy for patients with myocardial ischemia-reperfusion injury.

This is an experimental study and you're randomly assigned to either an intervention group or a control group. If you are assigned to the intervention group, you will receive an external shock therapy intervention on day 3 after coronary stenting in conjunction with currently optimal coronary drug therapy. Please do not display or share extracorporeal shock wave therapy to patients after coronary stent implantation. On the other hand, if you are assigned to the control group, you will receive the best available coronary medication. All participants will be invited to complete health examinations and questionnaires at baseline, at 1, 4, 6 ,12and 24 months after the intervention.

Your participation in this study is voluntary. If you decide not to participate or withdraw from the study, you may do so at any time without losing medical care or any other available treatment for your disease or condition. Any information obtained in this study will be kept strictly confidential and participants' names will not appear in any reports in this study at any time. Please feel free to contact Xianbin Li at 18469110649 or kmykdx@yeah.net with any questions or clarification regarding this study.

Thank you for your support and participation in this study.

Yours faithfully,

Li Xianbin, Master student,

The First Affiliated Hospital of Kunming Medical University

**Appendix B. Informed Consent form**

**Clinical efficacy of extracorporeal cardiac shock wave therapy for myocardial ischemia-reperfusion injury: a prospective randomized controlled trial**

By signing this consent form, I agree to participate in a study entitled **" Clinical efficacy of extracorporeal cardiac shock wave therapy for myocardial ischemia-reperfusion injury: a prospective randomized controlled trial"** conducted by Mr. Xianbin Li, a master's student at the First Affiliated Hospital of Kunming Medical University.

I understand that this study will be based on experimental methods and that I will be randomly assigned to a trial or control group. If I am selected for the trial group, in addition to the best coronary artery disease medication provided by the hospital, I will also receive free external cardiac shock therapy intervention provided by the hospital within 3 months after coronary artery stenting. I was told not to spread information about the intervention and not to present and discuss extracorporeal cardiac shock therapy to "other patients with coronary heart disease." I will be required to undergo regular external shock cardiotherapy interventions for 3 months after coronary stenting, and complete health examinations and questionnaires at baseline, 1, 4, 6, 12 and 24 months after the intervention. On the other hand, if I am assigned to the control group, I will receive the best medical treatment for coronary heart disease from the hospital. In addition, I will be required to complete the questionnaire independently.

I have been informed that all information that I provided will be kept confidential and will not be given to anyone else. I was guaranteed that all information I provided will be used only for research purposes and will be destroyed after five years from the study completion. I am aware that my participation is voluntary and I am free to withdraw from this study anytime without penalty of any kind.

I confirmed that I have read and have been informed of the study purpose, the procedure that will undergo and, the risk and benefit that I may experience. Alternatives to my participation have also been discussed. All my questions and personal concerns on all the above issues have been asked and fully explained by the researcher. I have read and understood this consent form. Therefore, I agree to give my consent to participate in this study.

Name of the participant:…………………………………………………………………

Signature of the participant:………….………….Date:………………………………...

Name of research personnel: ……………………………………………………………

Signature of research personnel:…………………Date:……………………………......

**Appendix C. Flow diagram of the study protocol**

**
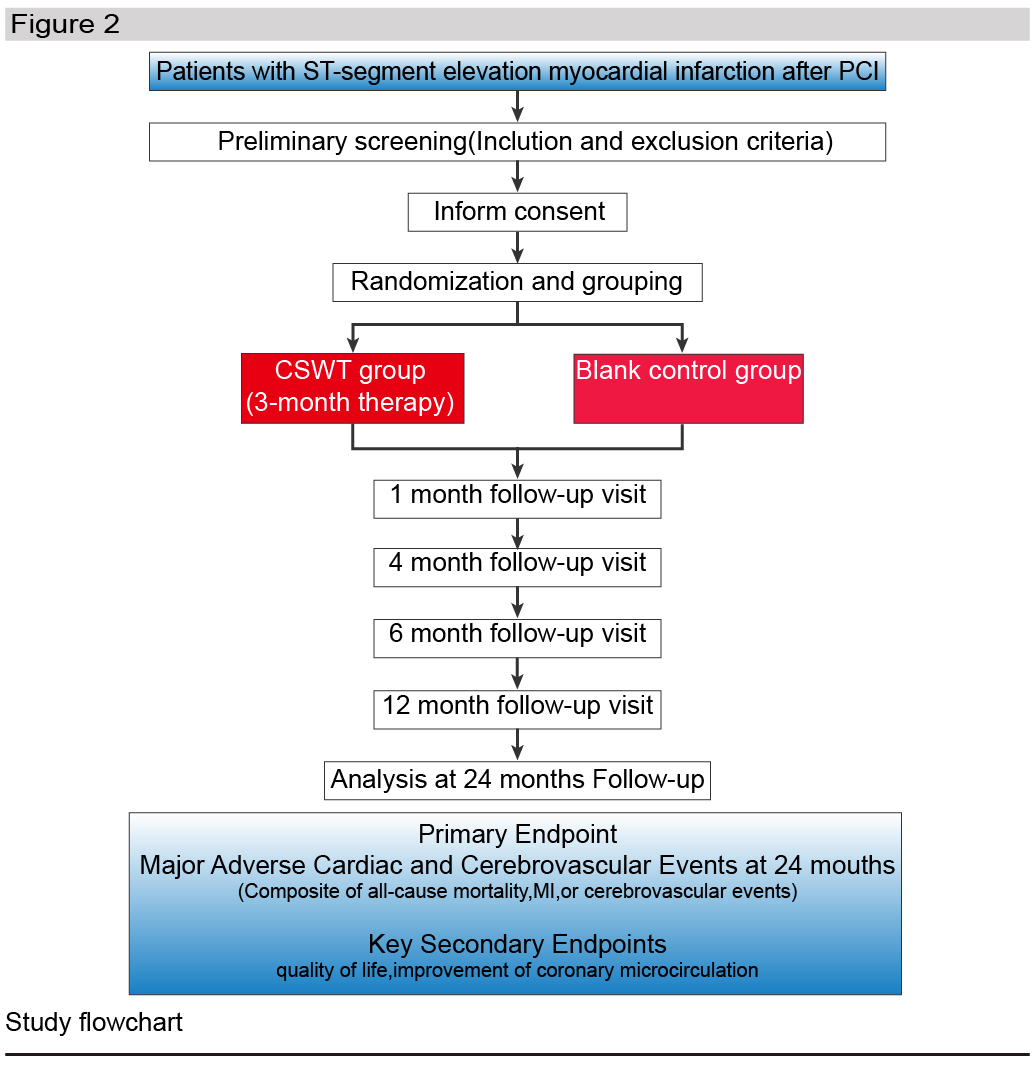
**

**Appendix D. Study questionnaire**

**The SF-8 rating scale**

|  |  |  |
| --- | --- | --- |

Date: Serial No

1. In general, in the past 4 weeks, do you think your general health status is:

□ excellent □ Very good □ good □ In general □ poor □ Very poor

2. In the past 4 weeks, has your physical condition limited your ability to do general physical activities (such as walking, climbing stairs, etc.)?

□ No restrictions at all □ A little bit limited □ There are some limitations

□ Very limited □ No physical activity

3. During the past 4 weeks, have you had any health problems carrying out your daily tasks, including going to work and doing housework?

□ It's not difficult at all □ Have a little difficulty □ Have some difficulties

□ Have great difficulty □ Unable to perform routine tasks

4. Have you had any physical pain in the last 4 weeks?

□ No pain at all □ I have a slight pain □ Have a slight pain

□ Have moderate pain □ Have severe pain □ I have very severe pain

5. Have you felt energetic in the past 4 weeks?

□ good □ better □ In general □ poor □ Very poor

6. During the past 4 weeks, to what extent have physical or mental health problems affected your social activities with family and friends?

□ Have no effect at all □ Have little effect □ Have some effect

□ Have a great influence □ You can't socialize

7. In the past 4 weeks, have you been troubled by emotional factors such as anxiety, depression, or irritability?

□ Doesn't bother me at all □ Have mild distress □ Have moderate distress

□ Have a great problem □ Extremely troubled

8. Have you had any personal or emotional problems during the past 4 weeks that have prevented you from carrying out your daily work, school, or other activities?

□ It doesn't affect it at all □ Have a little influence □ Have some effect

□ Have a great influence □ Unable to perform daily activities

**Seattle Angina Scale scoring Scale**

9. In the past 4 weeks, the following limitations were due to chest pain, chest compression and angina:

| activity | Severe restriction | Moderate limitation | Mild limitation | Slightly limited | unrestricted | Limited for other reasons |
| --- | --- | --- | --- | --- | --- | --- |
| Dress oneself | □ | □ | □ | □ | □ | □ |
| Indoor walking | □ | □ | □ | □ | □ | □ |
| shower | □ | □ | □ | □ | □ | □ |
| Climbing or stairs (three floors, non-stop) | □ | □ | □ | □ | □ | □ |
| Do outdoor activities or carry sundries | □ | □ | □ | □ | □ | □ |
| A brisk walk (one kilometer) | □ | □ | □ | □ | □ | □ |
| Jogging (1 km) | □ | □ | □ | □ | □ | □ |
| Lift or move heavy objects | □ | □ | □ | □ | □ | □ |
| Strenuous exercise (e.g. swimming or playing ball) | □ | □ | □ | □ | □ | □ |

10. Episodes of chest pain, chest compression, and angina during maximum intensity activity compared to 4 weeks ago:

□ Significantly increase □ Slight increase □ The same

□ Slight reduction □ Significantly reduce

11. Average number of episodes of chest pain, chest compression, and angina in the past 4 weeks:

□≥4 times/day □ 1-3 times/day □ ≥3 times/week

□ 1-2 times per week □ <1 times/week □ nonseizure

12. Average number of nitrodrugs (e.g. Nitroglycerin, analgesia, etc.) taken for chest pain, chest compression and angina in the past 4 weeks:

□ ≥4 times/day □ 1-3 times/day □≥3 times/week

□ 1-2 times per week □ <1 times/week □ Out of use

13. Trouble with following prescribed medication for chest pain, chest compression, and angina:

□ serious □ moderate □ slight □ Very few

□ There is no □ The doctor did not administer the medicine

14. Satisfaction with various measures for the treatment of chest pain, chest compression and angina:

□ Not satisfied □ Most dissatisfied □ Partial satisfaction

□ Most satisfied □ Highly satisfied

15. Satisfaction with doctor's explanation of chest pain, chest compression and angina:

□ Not satisfied □ Most dissatisfied □ Partial satisfaction

□ Most satisfied □ Highly satisfied

16. Overall, satisfaction with the current treatment of chest pain, chest compression and angina:

□ Not satisfied □ Most dissatisfied □ Partial satisfaction

□ Most satisfied □ Highly satisfied

17. Extent to which joy of living has been affected by chest pain, chest compression and angina in the past 4 weeks:

□ serious □ moderate □ slight □ Very few □ There is no

18. How would you feel if you still have chest pain, chest compression and angina in your future life?

□ Not satisfied □ Most dissatisfied □ Partial satisfaction

□ Most satisfied □ Highly satisfied

19. Heart attack and sudden death concerns:

□ Have been worried about □ Always worry about □ Sometimes worry □ Seldom worry □ Never worry
