# Supplementary material for: Principle and design of clinical efficacy observation of extracorporeal cardiac shock wave therapy for patients with myocardial ischemia-reperfusion injury: A prospective randomized controlled trial protocol
Source: PLoS One. 2023 Dec 8;18(12):e0294060. doi: 10.1371/journal.pone.0294060 (PMC10707494; doi:10.1371/journal.pone.0294060)
Supplement: S2 File — (PDF) [file pone.0294060.s003.pdf]

22

项目批准号	82260087
申请代码	H0212
归口管理部门	
依托单位代码	65003108A0616-1140

822600871004188

国家自然科学基金

资助项目计划书

（预算制项目）

资助类别：地区科学基金项目

亚类说明：

附注说明：

项目名称：体外心脏震波诱导外泌体源miR-140-3p拮抗心肌缺血再灌注血管内皮损伤的作用机制研究

直接费用：33万元　　执行年限：2023.01-2026.12

负 责 人：蔡红雁

通讯地址：云南省昆明市呈贡新城雨花街道春融西路1168号

邮政编码：650500　　电　　话：13888982853

电子邮件：hyflykm@sina.com

依托单位：昆明医科大学

联 系 人：游顶云　　电　　话：087165922623

填表日期：2022年09月19日

国家自然科学基金委员会制

Version：1.004.188

国家自然科学基金资助项目计划书填报说明
（预算制项目）

一、项目负责人收到《国家自然科学基金资助项目批准通知》（以下简称《批准通知》）后，请认真阅读本填报说明，参照国家自然科学基金相关项目管理办法和新修订的《国家自然科学基金资助项目资金管理办法》（以下简称《资金管理办法》，请查阅国家自然科学基金委员会官方网站首页“政策法规”栏目），按《批准通知》的要求认真填写和提交《国家自然科学基金资助项目计划书》（以下简称《计划书》）。

二、填写《计划书》时要科学严谨、实事求是、表述清晰、准确。《计划书》经国家自然科学基金委员会相关项目管理部门审核批准后，将作为项目研究计划执行、检查和验收的依据。

三、《计划书》各部分填写要求如下：

（一）简表：由系统自动生成。

（二）摘要及关键词：各类获资助项目都应当填写中、英文摘要及关键词。

（三）项目组主要成员：计划书中列出姓名的项目组主要成员由系统自动生成，与申请书原成员保持一致，不可随意调整。如果《批准通知》所附“项目评审意见及修改意见表”中“修改意见”栏目有调整项目组成员相关要求的，待项目开始执行后，按照项目成员变更程序另行办理。

（四）资金预算表：根据批准的项目资助额度，按规定调整项目预算，并按照《国家自然科学基金项目计划书预算表编制说明》填报资金预算表和预算说明书。

（五）正文：

1. 面上项目、地区科学基金项目：如果《批准通知》所附“项目评审意见及修改意见表”中“修改意见”栏目没有修改要求的，只需选择“研究内容和研究目标按照申请书执行”即可；如果《批准通知》中上述栏目明确要求调整研究期限或研究内容等的，须选择“根据研究方案修改意见更改”并填报相关修改内容。

2. 重点项目、重点国际（地区）合作研究项目、重大项目、国家重大科研仪器研制项目、原创探索计划项目：须选择“根据研究方案修改意见更改”，根据《批准通知》的要求填写研究（研制）内容，不得自行降低、更改研究目标（或仪器研制的技术性能与主要技术指标、验收技术指标等）或缩减研究（研制）内容。此外，还要突出以下几点：

   （1）研究的难点和在实施过程中可能遇到的问题（或仪器研制风险），拟采用的研究（研制）方案和技术路线；

   （2）项目主要参与者分工，合作研究单位（如有）之间的关系与分工，重大项目还需说明课题之间的关联；

   （3）详细的年度研究（研制）计划。

3. 创新研究群体项目：须选择“根据研究方案修改意见更改”，按下列提纲撰写：

   （1）研究方向；

（2）结合国内外研究现状，说明研究工作的学术思想和科学意义（限两个页面）；
（3）研究内容、研究方案及预期目标（限两个页面）；
（4）年度研究计划；
（5）研究队伍的组成情况。

4. 基础科学中心项目：须选择“根据研究方案修改意见更改”，根据《批准通知》的要求和现场考察专家组的意见和建议，进一步完善并细化研究计划，按下列提纲撰写：
（1）五年拟开展的研究工作（包括主要研究方向、关键科学问题与研究内容）；
（2）研究方案（包括骨干成员之间的分工及合作方式、学科交叉融合研究计划等）；
（3）年度研究计划；
（4）五年预期目标和可能取得的重大突破等；
（5）研究队伍的组成情况。

5. 对于其他类型项目，参照面上项目的方式进行选择和填写。

简表

<table>
<tr><td rowspan="6">项目负责人信息</td><td>姓　　名</td><td>蔡红雁</td><td>性 别</td><td>女</td><td>出生年月</td><td>1971年09月</td><td>民 族</td><td>汉族</td></tr>
<tr><td>学　　位</td><td colspan="3">博士</td><td>职称</td><td colspan="3">主任医师</td></tr>
<tr><td>是否在站博士后</td><td colspan="2">否</td><td colspan="2">电子邮件</td><td colspan="3">hyflykm@sina.com</td></tr>
<tr><td>电　　话</td><td colspan="2">13888982853</td><td colspan="2">个人网页</td><td colspan="3"></td></tr>
<tr><td>工 作 单 位</td><td colspan="7">昆明医科大学</td></tr>
<tr><td>所 在 院 系 所</td><td colspan="7">第一附属医院</td></tr>
<tr><td rowspan="3">依托单位信息</td><td>名　　称</td><td colspan="5">昆明医科大学</td><td>代码</td><td>65003108A0616</td></tr>
<tr><td>联 系 人</td><td colspan="2">游顶云</td><td colspan="2">电子邮件</td><td colspan="3">kykych@163.com</td></tr>
<tr><td>电　　话</td><td colspan="2">087165922623</td><td colspan="2">网站地址</td><td colspan="3"></td></tr>
<tr><td rowspan="2">合作单位信息</td><td colspan="8">单 位 名 称</td></tr>
<tr><td colspan="8"></td></tr>
<tr><td rowspan="7">项目基本信息</td><td>项 目 名 称</td><td colspan="7">体外心脏震波诱导外泌体源miR-140-3p拮抗心肌缺血再灌注血管内皮损伤的作用机制研究</td></tr>
<tr><td>资 助 类 别</td><td colspan="3">地区科学基金项目</td><td colspan="2">亚 类 说 明</td><td colspan="2"></td></tr>
<tr><td>附 注 说 明</td><td colspan="7"></td></tr>
<tr><td>申 请 代 码</td><td colspan="3">H0212:血管损伤、修复、重构和再生</td><td colspan="4">H0220:循环系统疾病研究新技术与新方法</td></tr>
<tr><td>基 地 类 别</td><td colspan="7"></td></tr>
<tr><td>执 行 年 限</td><td colspan="7">2023.01-2026.12</td></tr>
<tr><td>直 接 费 用</td><td colspan="7">33万元</td></tr>
</table>

项目摘要

中文摘要：

心肌缺血再灌注（MIR）引发的血管内皮损伤是影响急性心肌梗死患者预后的关键，但缺乏明确有效的防治措施。本项目前期研究发现体外心脏震波（ECSW）可诱导内皮祖细胞（EPCs）释放功能性外泌体，显著减轻心肌细胞缺氧/复氧损伤，但机制不清。进一步研究发现，ECSW能提高EPCs外泌体miR-140-3p的浓度，影响PTEN/HIF-1α信号通路，上调VEGF表达。已知VEGF、eNOS是改善MIR血管内皮功能的效应分子，但与miR-140-3p间的调控作用未见报道。我们推测：ECSW诱导EPCs-exo可能通过调控miR-140-3p/PTEN/HIF-1α轴，激活VEGF、eNOS表达，拮抗血管内皮损伤，达到防治MIR的作用。项目拟从临床、动物和细胞分子三个层面揭示ECSW调控EPCs-exo拮抗MIR血管内皮损伤的作用机制，为临床运用ECSW防治MIR损伤提供新的理论依据及作用靶点。

Abstract:

Vascular endothelial injury caused by myocardial ischemia-reperfusion (MIR) plays a key role in the prognosis of patients with acute myocardial infarction, but there is a lack of effective measures of prevention and treatment. In this project, we found that extracorporeal cardiac shock wave (ECSW) can induce endothelial progenitor cells (EPCs) to release functional exosomes (EPCs-exo), which can significantly reduce the hypoxia/reoxygenation injury of cardiomyocytes, but the mechanism was unclear. Further studies revealed that ECSW increased the concentration of exosomal miR-140-3p in EPCs, affected the PTEN/HIF-1α signaling pathway, and upregulated VEGF expression. VEGF and eNOS are known to be effector molecules to improve vascular endothelial function in MIR, but the regulatory interaction with miR-140-3p has not been reported. We hypothesize that ECSW-induced EPCs-exo could regulate the miR-140-3p/PTEN/HIF-1α axis, then activate VEGF, eNOS expression, thereby antagonizing vascular endothelial injury and achieving the effect of preventing MIR injury. This project aims to reveal the mechanism of ECSW by regulating EPCs-exo to antagonize MIR vascular endothelial injury on the levels of clinical, animal and cellular molecule, and provides new theoretical basis for the clinical application and promotion of ECSW therapy against MIR injury.

关键词(用分号分开)： 心肌缺血再灌注；体外心脏震波；内皮祖细胞；外泌体；血管内皮损伤

Keywords(用分号分开)： myocardial ischemia-reperfusion; extracorporeal cardiac shock wave; endothelial progenitor cells; exosomes; vascular endothelial injury

项目组主要成员

编号	姓名	出生年月	性别	职称	学位	单位名称	电话	证件号码	项目分工	每年工作时间（月）
1	蔡红雁	1971.09	女	主任医师	博士	昆明医科大学	13888982853	530102197109261145	项目负责人	6
2	刘怡希	1987.08	女	主治医师	博士	昆明医科大学	13708480819	530102198708191825	细胞实验	8
3	马文芳	1990.07	女	主治医师	博士	昆明医科大学	13700634023	522427199007172021	细胞实验	8
4	马一铭	1989.12	男	主治医师	硕士	昆明医科大学	15198810061	530421198912220713	动物实验	8
5	史云科	1988.07	男	医师	硕士	昆明医科大学	18987883349	53010319880704291X	动物实验	8

总人数	高级	中级	初级	博士后	博士生	硕士生
8	1	3	1	0	1	2

国家自然科学基金预算制项目预算表

项目批准号：82260087　　　　项目负责人：蔡红雁　　　　金额单位：万元

序号	科目名称	金额
1	一、基金资助项目直接费用合计	33.0000
2	1、 设备费	0.0000
3	其中：设备购置费	0.0000
4	2、 业务费	26.2000
5	3、 劳务费	6.8000
6	二、 其他来源资金	0.0000
7	三、 合计	33.0000

注：请按照项目研究实际需要合理填写各科目预算金额。

预算说明书

（请按照《国家自然科学基金项目计划书预算表编制说明》等的有关要求，按照政策相符性、目标相关性和经济合理性原则，实事求是编制项目预算。填报时，直接费用应按设备费、业务费、劳务费三个类别填报，每个类别结合科研任务按支出用途进行说明。填报时，对单价≥50万元的设备详细说明，对单价＜50万元的设备费用分类说明，**对合作研究单位资质及资金外拨情况、自筹资金进行必要说明。**）

申请的项目直接费用为33万元（用于支出科研业务、实验材料、测试化验加工费、出版/文献/信息传播/知识产权事务费、差旅费、劳务费等）。全部经费将按科研需要，严格按规定使用。

一、设备费：无

二、业务费：共计26.2万元，占直接费用79.4%。

1.材料费：共计21.44万元。用于购买实验动物、实验试剂盒、抗体、模拟物及抑制剂、其他试剂、细胞培养液、一次性培养皿、离心管、移液枪头等。

（1）购买400只SD大鼠（50元/只，共2.00万元，昆明医科大学实验动物部），动物饲养费用约1.0万元。合计3.00万元。

（2）购买细胞培养相关材料，如细胞培养所需血清（500ml，0.75万元×2=1.50万元，Gibco）、去除外泌体胎牛血清（100ml，0.45万元，SBI）、细胞培养所需培养基（0.05万元，Gibco）、内皮祖细胞EGM-2MV培养基（0.30万元×5=1.50万元，Lonza）、青霉素/链霉素胰酶（100ml，0.05万元，美国Corning公司）、一次性培养皿、培养瓶（0.2万元，美国Corning公司）、移液枪头（0.1万元，科进）、离心管（0.1万元，科进）、封口膜（0.02万元，百鲨）等耗材，合计：3.97万元。

（3）购买实验试剂盒：外泌体提取试剂盒（0.40万元/试剂盒×3=1.20万元，Invitrogen）、逆转录试剂盒（0.20万元/试剂盒×2=0.40万元，thermo）、miRNA转染试剂盒（0.50万元/试剂盒×2=1.00万元，Invitrogen）、qPCR试剂盒（0.20万元/试剂盒×2=0.4万元，Invitrogen）、TUNEL试剂盒（0.65万元，罗氏）、TTC试剂盒（0.06万元/试剂盒×5=0.30万元，Solarbio）、Annexin V-FITC试剂盒（0.10万元，Solarbio）、ELISA试剂盒（0.20万元/试剂盒×3=0.60万元，Invitrogen）、PKH26细胞连接试剂盒（0.5万元，Solarbio）等，合计：5.15万元。

（4）购买western blot抗体及其他材料：PTEN、HIF-1α、VEGF、eNOS、Bcl-2、Bax、Caspase3等抗体（一抗0.40万元/试剂盒，二抗0.24万元/试剂盒，共3.44万元，CST）、SDS凝胶试剂盒（0.10万元×5=0.50万元，CST）、BSA（0.05万元，CST）、PVDF（0.40万元，CST）、glycine（0. 05万元，CST）、DIPA（0.05万元，CST）等，合计：4.49万元。

（5）购买MISSION miR-140-3p mimic及NC（0.30万元，sigma）、miRNA inhibitor及NC（0.30万元，sigma）、慢病毒载体构建和细胞转染相关试剂（1.9万元）、双荧光素酶报告基因试剂盒（0.30万元×2=0.60万元，索莱宝）等，合计：3.1万元。

（6）购买蛋白酶K（0.05万元，宝生物）、RNA酶（0.05万元，宝生物）、4%水合氯醛（0.05万元，索莱宝）、4%多聚甲醛（0.02万元，索莱宝）、3%双氧水（0.01万元，索莱宝）、PBS（0. 10万元，100元/瓶×10=0.10万元，Hyclone）、大鼠骨髓淋巴细胞分离液试剂盒（0.10万元×2=0.20万元，索莱宝）、二甲基亚砜 DMSO（0.05万元，索莱宝）等实验试剂，合计：0.53万元。

2.测试化验加工费：共计1.46万元。

扫描电镜检测费：0.06万元/次×6次=0.36万元；流式细胞检测费：0.004万元/次×200=0.80万元；纳米追踪检测费：0.03万元/次×10=0.3万元。

3.差旅/会议/国际合作与交流费：共计2.00万元。用于课题组成员参加与项目有关的学术会议、学术交流、实验合作的会议费/差旅费。

参加全国性学术交流 4人次：（1）上海2人次，3天，机票费 0.40 万元，住宿费 0.10万元，交通及餐费0.30万元，合计0.80万元。（2）广州2人次，3天，机票费0.50万元，住宿费0.20万元，交通及餐费0.50万元，合计1.20万元。

4.出版/文献/信息传播/知识产权事务费：共计2.50万元。用于论文版面费和印刷费，文献检索等费。发表文章3篇：发表高质量期刊论文3篇，版面费0.80万元/篇，合计0.80×3=2.40万元。资料费、文献检索费等0.10万元。

三、劳务费，共计6.8万元，占直接费用20.6%。

1.研究生劳务费：共计6.0万元，用于直接参加项目研究的研究生的劳务费用。包括1名博士、2名研究生，按每年10个工作月，500元/月，共4年，合计：500元/月×3人×10月×4 年=6.0万元。

2.专家咨询费：共计0.80万元。邀请专家对研究中遇到困难和问题进行咨询和指导等费用：

800元/次/人，5位专家，邀请2次，共计800元/次/人×5人×2次=0.80万元。

报告正文

研究内容和研究目标按照申请书执行。

国家自然科学基金项目负责人、依托单位承诺书

国家自然科学基金项目负责人承诺书

本人郑重承诺：我接受国家自然科学基金的资助，严格遵守中共中央办公厅、国务院办公厅《关于进一步加强科研诚信建设的若干意见》《关于进一步弘扬科学家精神加强作风和学风建设的意见》《关于加强科技伦理治理的意见》等规定，及国家自然科学基金委员会关于资助项目管理、项目资金管理等各项规章，在《计划书》填写及项目执行过程中：

（一）按照《批准通知》《国家自然科学基金资助项目计划书填报说明》的要求填写《计划书》，未自行降低、更改目标任务或约定要求，或缩减研究（研制）内容；

（二）树立“红线”意识，严格履行科研合同义务，按照《计划书》负责实施本项目（批准号：82260087），切实保证研究工作时间，按时报送有关材料，及时报告重大情况变动，不违规将科研任务转包、分包他人，不以项目实施周期外或不相关成果充抵交差；

（三）遵守科研诚信、科技伦理规范和学术道德，认真开展研究工作，对资助项目发表的论著和取得的研究成果按规定进行标注，不在非本项目资助的成果或其他无关成果上标注本项目批准号，反对无实质学术贡献者“挂名”，不在成果署名、知识产权归属等方面侵占他人合法权益，并如实报告本人及项目组成员发生的违背科研诚信要求的任何行为；

（四）尊重科研规律，弘扬科学家精神，严谨求实，追求卓越，反对浮夸浮躁、投机取巧，不人为夸大学术或技术价值，不传播未经科学验证的现象和观点；

（五）将项目资金全部用于与本项目研究工作相关的支出，并结合科研活动需要，科学合理安排项目资金支出进度；

（六）做好项目组成员的教育和管理，确保遵守以上相关要求。

如违背上述承诺，本人愿接受国家自然科学基金委员会和相关部门做出的各项处理决定。

项目负责人（签字）：李红雁

2022年 10月 8日

依托单位科研管理部门：	依托单位财务管理部门：
负责人（签章）：徐宏慧<br>2022年 10月 17日	负责人（签章）：李瑞民<br>2022年 10月 17日

国家自然科学基金项目依托单位承诺书

我单位同意承担上述国家自然科学基金项目，将保证项目负责人及其研究队伍的稳定和研究项目实施所需的条件，严格遵守国家自然科学基金委员会有关资助项目管理、项目资金管理、科研诚信管理和科技伦理管理等各项规定，并督促实施。

依托单位（公章）

2022年 10月 17日

国家自然科学基金资助项目签批审核表

<table>
<tr><td rowspan="2">本栏目由自然科学基金委填写</td><td>科学处审查意见：

同意按计划执行

王 疏
负责人（签章）：
年 月 日
2023年2月13日</td></tr>
<tr><td>科学部审查意见：

同意科学处意见

负责人（签章）：
年 月 日 闫章才
2023年2月13日</td></tr>
</table>

项目名称： 体外心脏震波诱导外泌体源miR-140-3p拮抗心肌缺血再灌注血管内皮损伤的作用机制研究

资助类型： 地区科学基金项目

申请代码： H0212.血管损伤、修复、重构和再生

国家自然科学基金项目申请人和参与者承诺书

为了维护国家自然科学基金项目评审公平、公正，共同营造风清气正的科研生态，本人**在此郑重承诺：**严格遵守《中华人民共和国科学技术进步法》《国家自然科学基金条例》《关于进一步加强科研诚信建设的若干意见》《关于进一步弘扬科学家精神加强作风和学风建设的意见》以及科技部、自然科学基金委关于科研诚信建设有关规定和要求；申请材料信息真实准确，不含任何涉密信息或敏感信息，不含任何违反法律法规或违反科研伦理规范的内容；在国家自然科学基金项目申请、评审和执行全过程中，恪守职业规范和科学道德，遵守评审规则和工作纪律，杜绝以下行为：

（一）抄袭、剽窃他人申请书、论文等科研成果或者伪造、篡改研究数据、研究结论；

（二）购买、代写申请书；购买、代写、代投论文，虚构同行评议专家及评议意见；购买实验数据；

（三）违反成果发表规范、署名规范、引用规范，擅自标注或虚假标注获得科技计划等资助；

（四）在项目申请书中以高指标通过评审，在项目计划书中故意篡改降低相应指标；

（五）以任何形式探听或散布尚未公布的评审专家名单及其他评审过程中的保密信息；

（六）本人或委托他人通过各种方式和途径联系有关专家进行请托、游说，违规到评审会议驻地窥探、游说、询问等干扰评审或可能影响评审公正性的行为；

（七）向工作人员、评审专家等提供任何形式的礼品、礼金、有价证券、支付凭证、商业预付卡、电子红包，或提供宴请、旅游、娱乐健身等任何可能影响评审公正性的活动；

（八）违反财经纪律和相关管理规定的行为；

（九）其他弄虚作假行为。

如违背上述承诺，本人愿接受国家自然科学基金委员会和相关部门做出的各项处理决定，包括但不限于撤销科学基金资助项目，追回项目资助经费，向社会通报违规情况，取消一定期限国家自然科学基金项目申请资格，记入科研诚信严重失信行为数据库以及接受相应的党纪政务处分等。

申请人签字：李红雁

编号	参与者姓名 / 工作单位名称（应与加盖公章一致）/ 证件号码	签字
1	**刘怡希** / 昆明医科大学 / 5**************5	刘怡希
2	**马文芳** / 昆明医科大学 / 5**************1	马文芳
3	**马一铭** / 昆明医科大学 / 5**************3	马一铭
4	**史云科** / 昆明医科大学 / 5**************X	史云科
5		
6		
7		
8		
9		

项目名称： 体外心脏震波诱导外泌体源miR-140-3p拮抗心肌缺血再灌注血管内皮损伤的作用机制研究

资助类型： 地区科学基金项目

申请代码： H0212.血管损伤、修复、重构和再生

国家自然科学基金项目申请单位承诺书

为了维护国家自然科学基金项目评审公平、公正，共同营造风清气正的科研生态，**本单位郑重承诺：**申请材料中不存在违背《中华人民共和国科学技术进步法》《国家自然科学基金条例》《关于进一步加强科研诚信建设的若干意见》《关于进一步弘扬科学家精神加强作风和学风建设的意见》以及科技部、自然科学基金委关于科研诚信建设有关规定和要求的情况；申请材料符合《中华人民共和国保守国家秘密法》和《科学技术保密规定》等有关法律法规和规章制度要求，不含任何涉密信息或敏感信息；申请材料不含任何违反法律法规或违反科研伦理规范的内容；申请人符合相应项目的申请资格；在项目申请和评审活动全过程中，遵守有关评审规则和工作纪律，杜绝以下行为：

（一）以任何形式探听或公布未公开的项目评审信息、评审专家信息及其他评审过程中的保密信息，干扰评审专家的评审工作；

（二）组织或协助申请人/参与者向工作人员、评审专家等给予任何形式的礼品、礼金、有价证券、支付凭证、商业预付卡、电子红包等；宴请工作人员、评审专家，或组织任何可能影响科学基金评审公正性的活动；

（三）支持、放任或对申请人/参与者抄袭、剽窃、重复申报、提供虚假信息（含身份和学术信息）等不当手段申报国家自然科学基金项目疏于管理；

（四）支持或协助申请人/参与者采取“打招呼”“围会”等方式影响科学基金项目评审；

（五）其他违反财经纪律和相关管理规定的行为。

如违背上述承诺，本单位愿接受自然科学基金委和相关部门做出的各项处理决定，包括但不限于停拨或核减经费、追回项目已拨经费、取消本单位一定期限国家自然科学基金项目申请资格、记入科研诚信严重失信行为数据库以及主要责任人接受相应党纪政务处分等。

依托单位公章：

日期： 2022年 10月 17日

合作研究单位公章：

日期： 年 月 日

合作研究单位公章：

日期： 年 月 日
