# Supplementary material for: Principle and design of clinical efficacy observation of extracorporeal cardiac shock wave therapy for patients with myocardial ischemia-reperfusion injury: A prospective randomized controlled trial protocol
Source: PLoS One. 2023 Dec 8;18(12):e0294060. doi: 10.1371/journal.pone.0294060 (PMC10707494; doi:10.1371/journal.pone.0294060)
Supplement: S3 File — (PDF) [file pone.0294060.s004.pdf]

昆明医科大学第一附属医院项目申报伦理委员会同意证明

申报项目名称	体外心脏震波诱导外泌体源miR-140-3p拮抗心肌缺血再灌注血管内皮损伤的作用机制	
项目负责人	蔡红雁	
联系电话	13888982853	
申报项目类别	国家自然科学基金	
	选择其他项目请填写：	
项目类型	是否涉及临床诊疗使用超范围、超说明书或未上市药品：	○ 是 ◉ 否
	是否涉及临床诊疗使用未上市医疗器械：	○ 是 ◉ 否
	是否涉及临床使用超出诊疗规范的新技术：	○ 是 ◉ 否
研究方案	研究方案.docx	
科技教育部审查意见	经形式审查，该项目为心内科蔡红雁拟申报的2022年国家自然科学基金项目，按照科研项目管理规定，研究方案须经伦理委员会伦理审查通过后，方能进行项目申报，报领导审批。<br>颜光前 科技教育部 (2022-03-06 22:10)<br>拟同意报伦理审查，报请院领导审批。<br>科技教育部 (2022-03-07 09:42)<br>同意<br>院务部 (2022-03-07 09:42)	
研究方案审查意见	同意<br>院务部 (2022-03-07 18:10)<br>同意<br>院务部 (2022-03-07 18:18)	
伦理委员会主任委员意见	同意<br>党委办公室 (2022-03-08 09:40)	

伦理委员会
