# Supplementary material for: Principle and design of clinical efficacy observation of extracorporeal cardiac shock wave therapy for patients with myocardial ischemia-reperfusion injury: A prospective randomized controlled trial protocol
Source: PLoS One. 2023 Dec 8;18(12):e0294060. doi: 10.1371/journal.pone.0294060 (PMC10707494; doi:10.1371/journal.pone.0294060)
Supplement: Supplementary file 5 [file pone.0294060.s005.doc]

Approval
certificate of Project Application Ethics Committee of the First Affiliated
Hospital of Kunming Medical University

|  |  |  |
| --- | --- | --- |
| Name of application project | Mechanism of Extracorporeal cardiac shock wave induction of exosome derived miR-140-3p on endothelial injury induced by myocardial ischemia reperfusion | |
| Project leader | CAI Hongyan | |
| Contact number | +8613888982853 | |
| Category of declared items | National Natural Science Foundation of China | |
| Please fill in other items: |  |
| Item type | Whether it involves the use of out-of-range, off-label or unmarketed drugs in clinical treatment: | yes     no |
| Whether it involves the use of unlisted medical devices for clinical treatment: | yes     no |
| Whether it involves the clinical use of new technology beyond the diagnostic and treatment specifications: | yes     no |
| Research program | Research program.docx | |
| Review opinions of the Ministry of Science, Technology and Education | According to the form review, this project is the 2022 National Natural Science Foundation project that CAI Hongyan from the Department of Cardiology intends to apply for. According to the regulations on the management of scientific research projects, the project can only be applied and submitted to the leader for approval after the research proposal has been approved by the ethics committee.      Yan Guangqian  Ministry of Science, Technology and Education (2022-03-06 22:10)     I plan to submit to the ethical review for approval by the leaders of the hospital.     Tang Wei   Ministry of     Science, Technology and Education (2022-03-07 09:42)  agree    Liang Hongmin  Department of hospital (2022-03-07 09:42) | |
| Study program review comments | agree  Peng Yunzhu Department of hospital (2022-03-07 18:10)  agree  Zeng Zhong Department of hospital (2022-03-07 18:18) | |
| Comments of the Chairman of the Ethics Committee | agree  Zhou Jia  Party committee office (2022-03-08 09:40) | |
